# Dependence of Performance of Si Nanowire Solar Cells on Geometry of the Nanowires

**DOI:** 10.1155/2014/358408

**Published:** 2014-01-16

**Authors:** Firoz Khan, Seong-Ho Baek, Jae Hyun Kim

**Affiliations:** Energy Research Division, Daegu Gyeongbuk Institute of Science & Technology (DGIST), 50-1 Sang-Ri, Hyeonpung-Myeon, Dalseong-gun, Daegu 711-873, Republic of Korea

## Abstract

The dependence of performance of silicon nanowires (SiNWs) solar cells on the growth condition of the SiNWs has been described. Metal-assisted electroless etching (MAE) technique has been used to grow SiNWs array. Different concentration of aqueous solution containing AgNO_3_ and HF for Ag deposition is used. The diameter and density of SiNWs are found to be dependent on concentration of solution used for Ag deposition. The diameter and density of SiNWs have been used to calculate the filling ratio of the SINWs arrays. The filling ratio is increased with increase in AgNO_3_ concentration, whereas it is decreased with increase in HF concentration. The minimum reflectance value achieved is ~1% for SiNWs of length of ~1.2 **μ**m in the wavelength range of 300–1000 nm. The performance and diode parameters strongly depend on the geometry of SiNWs. The maximum short circuit current density achieved is 35.6 mA/cm^2^. The conversion efficiency of solar cell is 9.73% for SiNWs with length, diameter, and wire density of ~1.2 **μ**m, ~75 nm, and 90 **μ**m^−2^, respectively.

## 1. Introduction

The increasing demand for energy and the effect of global warming are two related issues attracting more and more attention from human being. Photovoltaic technology is the most promising approach to carbon-free energy production and it has received more attention [1]. Nanostructures can be used as solution for better photovoltaic application [2]. The photovoltaic based on silicon nanowires (SiNWs) are becoming more attractive due to their optical and electrical properties compared to bulk Si [3, 4]. The important role of SiNWs arrays is to reduce reflection losses in photovoltaic devices. The reduction in the reflectance of SiNWs arrays is due to improved optical impedance matching between air and Si [5, 6], multiple incident light scattering inside the arrays [7], and the role of individual SiNWs as nanoscale resonators with the antenna effect [8]. Several techniques have been reported in the literature to synthesize the SiNWs [9–11].

Several studies have been made to design the structural parameters, namely, filling ratio, wire density, and diameter, to enhance light absorption in SiNWs solar cells [12, 13]. Some of the researchers [14, 15] have done the simulation for diameter and periodicity ratio dependence on the optical absorption in the SiNWs arrays [16–18]. Previously, some of the groups [19–21] investigated that randomly distributed SiNWs array gives better absorption than the regularly distributed one due to stronger scattering. Metal-assisted electroless etching (MAE) technique has been recently developed for fabrication of randomly distributed SiNWs arrays [22–24]. It is important to design particular diameter and wire density (periodicity) to achieve minimum reflectance and hence to get the highest current density for SiNWs based solar cells. Secondly, reduction of the recombination and resistive losses are equally important to get the better conversion efficiency. The reflectance can be easily reduced by increasing the length of SiNWs but recombination and resistive losses also increase with the increase in SiNWs length. Thus, the SiNWs of smaller length with ultralow reflectance are potentially feasible for antireflection applications in next-generation solar cells [22–26]. Hence, it is important to optimize the geometrical parameters for obtaining SiNWs arrays as superior antireflective characteristics with minimum recombination and resistive losses. Recently, Jung et al. [27] have optimized Ag deposition time to get maximum conversion efficiency.

In this paper, we have tried to achieve minimum reflectance ~1% in the wavelength range 300–1000 nm by engineering the diameter and density of SiNWs of length ~1.2 *μ*m to get the minimum reflection, recombination and resistive losses.

## 2. Materials and Methods

### 2.1. Synthesis of SiNWs

SiNWs arrays were synthesized on Cz grown, boron doped p-type, 0.2–0.6 Ω cm resistivity, 550 mm thickness, and <100> oriented polished Si wafers using two steps metal-assisted wet etching (metal deposition and etching) method. First, the substrates were cleaned in hot solution of H_2_O_2_ and H_2_SO_4_ (1 : 3 by volume) for 10 min. Then, the substrates were dipped in dilute HF to remove oxide grown in the cleaning process. The silver nanoparticles (NPs) were uniformly deposited onto the Si substrate using an aqueous solution of AgNO_3_ and HF. Two types of solutions (A and B) were used separately for Ag deposition for two sets of experiments. In set A, the concentration of AgNO_3_ was taken as 0.01, 0.02, 0.03, and 0.04 M (i.e., set A_1_, A_2_, A_3_, and A_4_) and the concentration of HF was constant (4.6 M). In set B, the concentration of AgNO_3_ was constant (0.03 M) and the concentration of HF was taken as 2.3, 4.6, 6.9, and 9.2 M (i.e., set B_1_, B_2_, B_3_, and B_4_). Henceforth, we shall refer to the samples simply as A_1_, A_2_, A_3_, A_4_, B_1_, B_2_, B_3_, and B_4_ in the text. It can be noted that the samples A_3_ and B_2_ were identical because the solution concentration was same. In the Ag deposition process, the Ag ions captured electrons from Si and reduced to Ag metal NPs. These nanoparticles are deposited on the silicon surfaces. The areal density of Ag NPs depends upon solution concentration, deposition time, and temperature. The etching was performed at room temperature in a mixed solution of deionized water (DIW), HF (4.6 M), and H_2_O_2_ (0.5 M) to obtain a vertically aligned SiNWs array. After completing the electroless etching, SiNW samples were dipped in concentrated HNO_3_ for 10 min to completely remove the remaining Ag NPs, then rinsed in DIW, and dried in air. The filling ratio was calculated using method of Jung et al. [27].

### 2.2. Solar Cell Fabrication

The SiNWs samples were subjected to RCA cleaning before the fabrication process. After RCA cleaning, the front and back sides were simultaneously diffused by phosphorous and boron using phosphorus-silicate and borosilicate glass to create n^+^-p-p^+^ structure by spin on dopant (SOD) technique. Phosphorus and boron silicate precursors (P509 for P and B200 for B, supplied by Filmtronics) were spin-coated on different dummy wafers. Then, the SiNWs samples were loaded between the dummy wafers in a conventional quartz-tube furnace. The diffusion was carried out at 1000°C for 5 min, while the target samples were kept at a closely spaced distance.

After diffusion, the phosphosilica glass (PSG) and borosilica glass (BSG) were removed simply by immersing the prepared specimens in buffer oxide etchant (BOE) for 10 min. The patterned front gridded metal contacts were made by Ti/Ag using metal mask. The back contacts were made using Ti/Ag on the full surface. The contacts were sintered in rapid thermal processing unit (RTP) at 600°C. Finally, edges of the solar cells were scribed for isolation of junction.

### 2.3. Characterization Techniques

The field emission scanning electron microscopy (FE-SEM) images were taken for front and cross-sectional views of SiNWs samples using Hitachi FE-SEM Model S-4800. The reflectance of SiNWs samples was measured in the wavelength range of 300–1200 nm with Perkin-Elmer Model Lambda 750 using an integrated sphere.

The illuminated current density-voltage (*J*-*V*) characteristics of solar cells were measured under 100 mW/cm^2^ intensity with AM1.5 Global spectrum using a Keithley sourcemeter Model 2400 (with basic accuracy 0.012%) and Newport 91192 solar simulator system (equipped with 1KW Xenon arc lamp from Oriel). The illumination intensity was measured using a reference silicon solar cell obtained from PV Measurements, USA. All the measurements were carried out at 25°C.

## 3. Results and Discussion

### 3.1. Geometry of SiNWs Array

**Figure 1:**
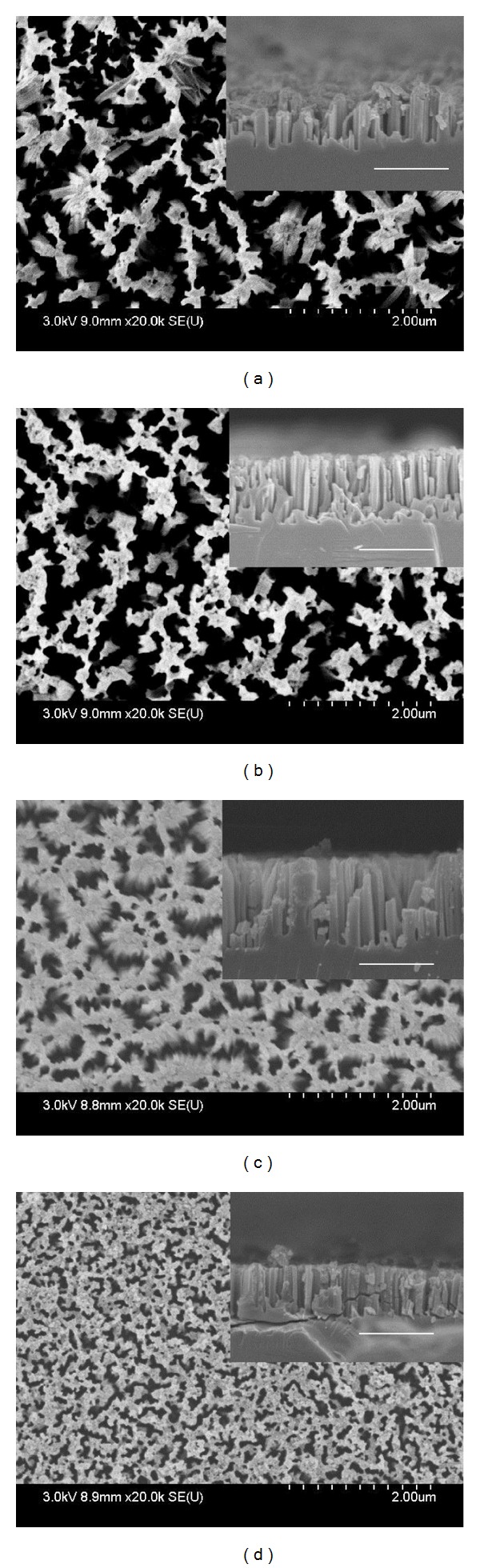
FE-SEM images of front and cross-sectional (in inset) view of SiNWs array grown using solutions (a) A_1_, (b) A_2_, (c) A_3_, and (d) A_4_ for Ag deposition.

**Figure 2:**
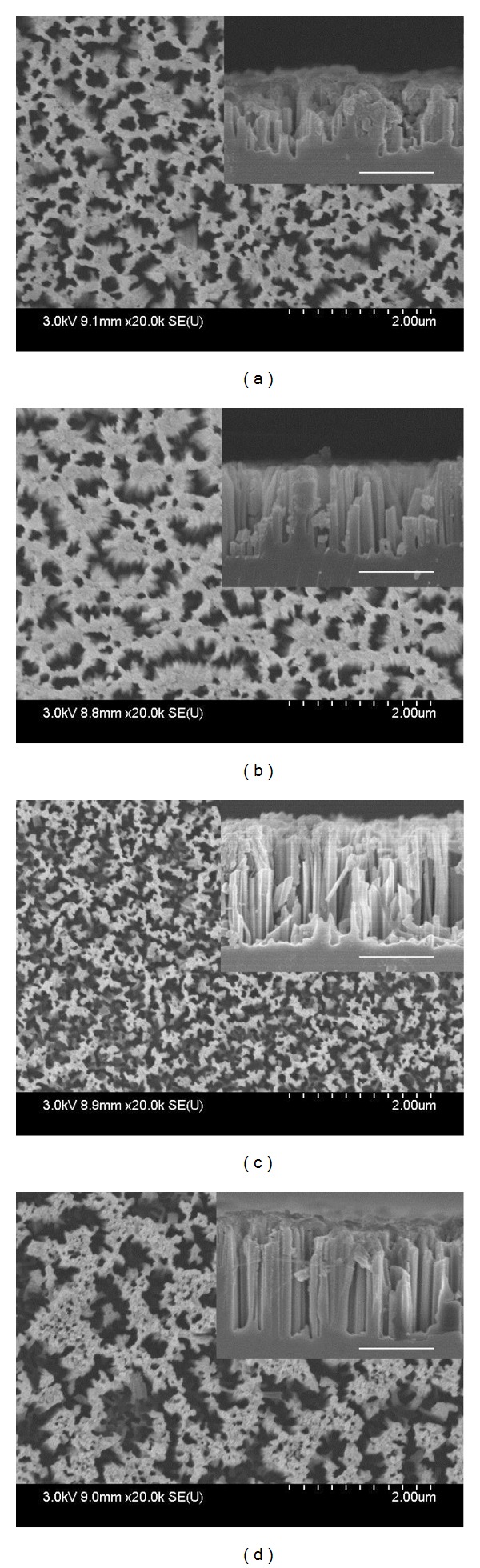
FE-SEM images of front and cross-sectional (in inset) view of SiNWs array grown using solutions (a) B_1_, (b) B_2_, (c) B_3_, and (d) B_4_ for Ag deposition.

**Figure 3:**
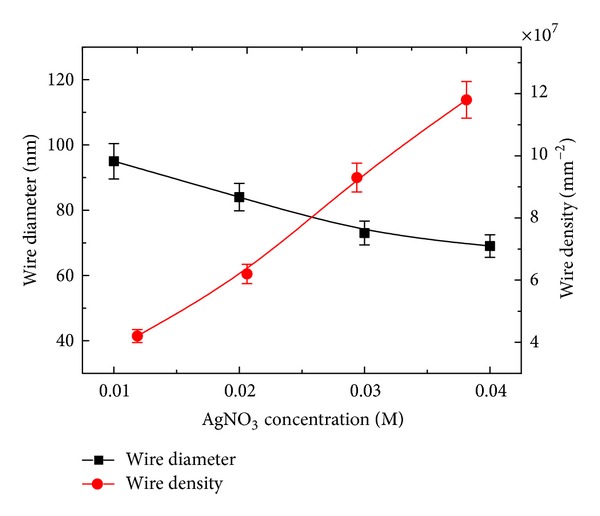
Variation of average diameters and density of SiNWs with AgNO_3_ concentration.

**Figure 4:**
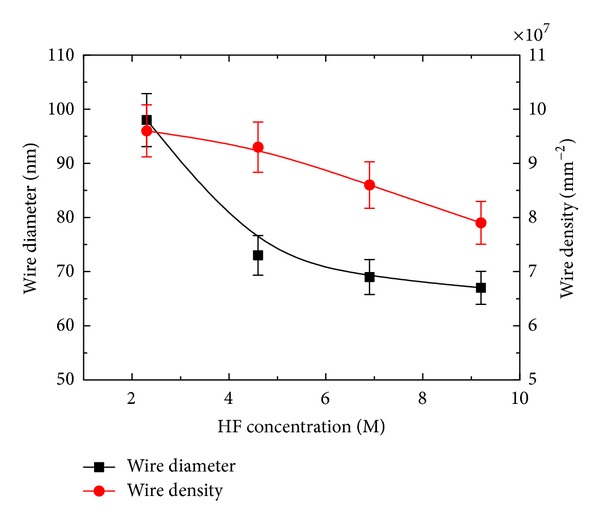
Variation of average diameter and density of SiNWs with HF concentration.

**Figure 5:**
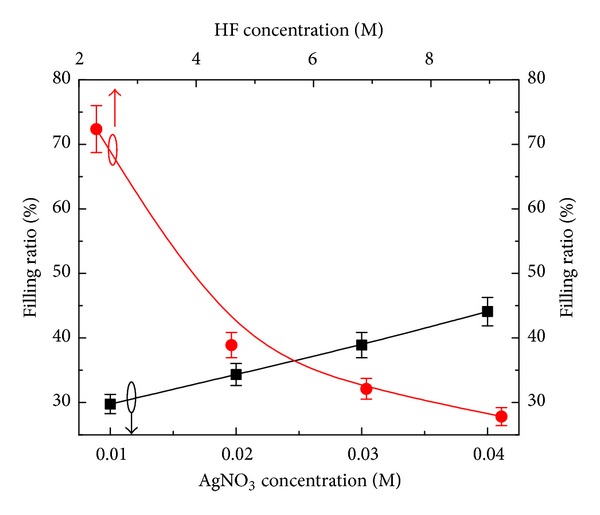
Dependence of filling ratio of SiNWs array with AgNO_3_ and HF concentrations.

The front and cross-sectional (in inset) views of SiNWs arrays of both sets are shown in Figures 1(a)–1(d) and Figures 2(a)–2(d), respectively. It can be seen from Figures 1(a)–1(d) that the surface morphology (number of SiNWs per unit area and diameter of SiNWs) is changing with the AgNO_3_ concentration. The diameter of SiNWs is decreased with increase in AgNO_3_ concentration, whereas the SiNWs density (number of SiNWs per unit area) is increased with increase in the AgNO_3_ concentration. The variation of SiNWs (wire) diameter and density is shown in Figure 3. The increase in wire density may be due to increase in number of Ag nanoparticles with increase in AgNO_3_ concentration. The reduction in wire diameter may be due to decrease in the Ag nanoparticles size with increase in AgNO_3_ concentration. The variation of wire diameter and density with HF concentrations is shown in Figure 4. Both the wire diameter and density are decreased with increase in HF concentration. The diameter of the wires is initially decreased with fast rate then becomes slower. The wire density is decreased linearly with increase in HF concentration. The value of diameters and densities of wires has been used to calculate the filling ratio of SiNWs arrays. The variation of filling ratio of SiNWs with AgNO_3_ and HF concentration are shown in Figure 5. The filling ratio is linearly increased with increase in AgNO_3_ concentration, whereas it is exponentially decreased with the increase in HF concentration.

### 3.2. Dependence of Reflectance of SiNWs Arrays on Growth Condition

**Figure 6:**
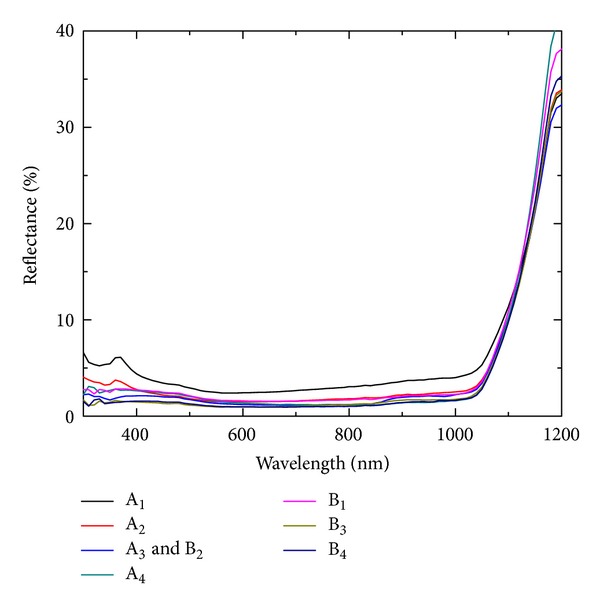
Reflectance spectra of SiNWs arrays grown, using different solutions for Ag deposition.

The reflectance spectra of all the samples are shown in Figure 6. The minimum reflectance of ~1% is observed for samples A_3_ and B_2_, B_3_, and B_4_ in the wavelength range 600–800 nm, whereas, in the wavelength range of 300–1000 nm, sample B_3_ has the lowest reflectance value. It can be noted that the minimum reflectance is obtained for filling ratio of ~40%. In case of samples B_3_ and B_4_, the length (~1.5 *μ*m) of nanowires is greater than in the all others samples (~1.2 *μ*m) for the same Ag deposition time and the same etching conditions. The highest (~2.43%) and the lowest (~0.93%) values of reflectance are obtained for the samples A_1_ and B_3_, respectively.

### 3.3. Analysis of Performance Parameters

**Figure 7:**
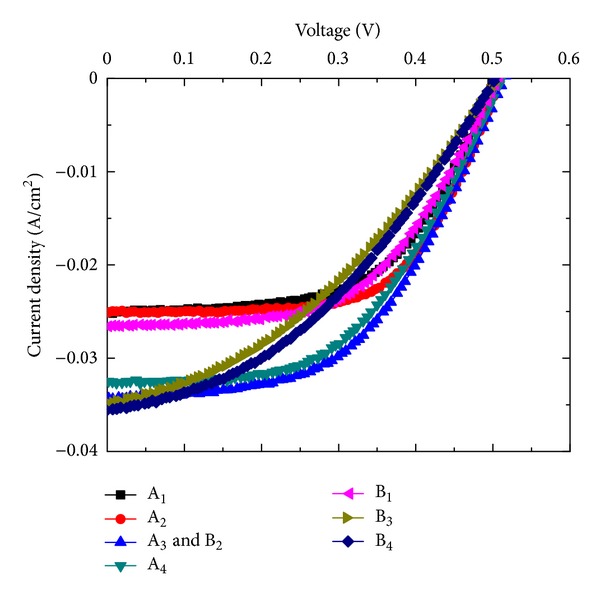
Illuminated *J*-*V* characteristics of solar cells with SiNWs grown, using different solutions for Ag deposition.

The illuminated *J*-*V* curves obtained at 25°C under illumination condition of 100 mW/cm^2^ of simulated AM1.5G solar radiation of the above samples are shown in Figure 7. It can be seen from Figure 7 the maximum value of conversion efficiency obtained for samples A_3_ and B_2_. The performance parameters, namely, short circuit current density *J*_sc_, open circuit voltage *V*_oc_, curve factor CF, and conversion efficiency *η* of the solar cells, are listed in Table 1. The current densities of samples B_3_ and B_4_ are higher than those of the samples A_3_ and B_2_ due to lowest reflection in the wavelength range of 300–1200 nm but conversion efficiencies are very low due to low CF values. The highest and the lowest *J*_sc_ values are obtained as 35.6 and 25.0 mA/cm^2^ for the samples A_1_ and B_4_, respectively. The maximum achieved *V*_oc_ value is 0.534 V for samples A_3_ and B_2_. The minimum and maximum CF values obtained are 0.357 and 0.597 for samples B_3_ and A_2_, respectively. A low CF value of sample B_3_ is mainly due to the lower value of shunt resistance (*R*_sh_). A lower value *V*_oc_ is obtained for the samples B_3_ and B_4_ due to the low value of *R*_sh_, high surface recombination velocity. The length of nanowires of samples B_3_ and B_4_ is higher (~1.5 *μ*m) than that of the other samples, so the surface recombination velocity is increased due to increase in active surface area (number of dangling bonds). The low CF values of the samples B_3_ and B_4_ are mainly due to high series resistance (*R*_*s*_). The highest conversion efficiency of 9.73% is obtained for samples A_3_ and B_2_.

### 3.4. Analysis of Diode Parameters

The values of *R*_sh_, *R*_*s*_, diode ideality factor (*n*), and reverse saturation current density (*J*_0_) are listed in Table 2. The *R*_sh_ values of samples B_3_ and B_4_ (45.72 and 55.31, resp.) are lower among all the samples (more than 200 Ω cm^2^) due to high porosity (low filling ratio). The low filling ratio is resulted to slightly puncture the junction that increased the shunt current. Due to larger surface area of the samples B_3_ and B_4_, the surface recombination is high which increased the values of *n* and *J*_0_. The *R*_*s*_ values of these two samples are the lowest among all the samples. The highest value of *R*_sh_ was obtained for sample A_2_. The *R*_*s*_ values for all the samples are found to be in between 2 and 3 Ω cm^2^. The lowest value of *R*_*s*_ (2.058 Ω cm^2^) was obtained for the sample B_3_. The *n* values of all the samples are found to be ~2 but, for the samples B_3_ and B_4_, the *n* value is very high (~5) due to high surface recombination. The *J*_0_ values for all the samples are found between 2.0 × 10^−7^ and 5.0 × 10^−4^ A/cm^2^. The lowest values of *n* and *J*_0_ have been obtained for sample A_2_ (1.788, 2.363 × 10^−7^ A/cm^2^), whereas highest values are obtained for sample B_3_ (5.356, 5.211 × 10^−4^ A/cm^2^), respectively.

## 4. Conclusions

Dependence of SiNWs solar cells performance on the concentration of Ag deposition solution has been investigated. The filling ratio of SiNWs array changed with AgNO_3_ and HF concentration. The diameter of SiNWs is decreased, whereas wire density is increased linearly with increase in AgNO_3_ concentration. Both the diameter and wire density are decreased with increase in HF concentration. The filling ratio of the SiNWs array is increased with increase in AgNO_3_ concentration, whereas it is decreased with increase in HF concentration. The minimum reflectance value of ~1% is achieved for the filling ratio of ~40%. It has been observed that the performance and diode parameters strongly depend on the solution concentration used for Ag nanoparticle deposition. A maximum short circuit current density of 35.6 mA/cm^2^ is obtained for the sample B_4_. The highest conversion efficiency of 9.73% is achieved with CF of 0.531 for aqueous solution of HF (4.6 M) and AgNO_3_ (0.03 M). The efficiency can be improved with improving the CF values using better process techniques.

## Figures and Tables

**Table 1:** The values of performance parameters obtained at 25°C under 100 mW/cm^2^ intensity of simulated AM1.5G solar radiation along with their reflectance at wavelength *λ* = 600 nm.

Sample	*R* (%) at *λ* = 600 nm	*J*_sc_ (A/cm^2^)	*V*_oc_ (V)	CF	*η* (%)
A_1_	2.43	0.0250	0.521	0.555	7.23
A_2_	1.51	0.0251	0.529	0.597	7.93
A_3_ & B_2_	1.22	0.0343	0.534	0.531	9.73
A_4_	1.41	0.0325	0.531	0.501	8.65
B_1_	1.62	0.0266	0.526	0.525	7.35
B_3_	0.93	0.0349	0.523	0.357	6.52
B_4_	0.94	0.0356	0.524	0.374	6.98

**Table 2:** The values of diode parameter (shunt resistance, series resistance, diode ideality factor, and reverse saturation current density) obtained at 25°C from illuminated *J*-*V* curves.

Sample	*R*_sh_ (Ω cm^2^)	*R*_*s*_ (Ω cm^2^)	*n *	*J*_0_ (A/cm^2^)
A_1_	251.88	2.222	2.364	4.276*E* − 6
A_2_	452.49	2.625	1.788	2.363*E* − 7
A_3_ & B_2_	204.92	2.736	2.713	1.479*E* − 5
A_4_	645.16	3.415	2.531	8.937*E* − 6
B_1_	380.22	3.329	2.444	5.749*E* − 6
B_3_	45.72	2.058	5.356	5.211*E* − 4
B_4_	55.31	2.190	5.040	4.575*E* − 4
